# Bioinformatics-based discovery of the urinary BBOX1 mRNA as a potential biomarker of diabetic kidney disease

**DOI:** 10.1186/s12967-019-1818-2

**Published:** 2019-02-28

**Authors:** Le-Ting Zhou, Lin-Li Lv, Shen Qiu, Qing Yin, Zuo-Lin Li, Tao-Tao Tang, Li-Hua Ni, Ye Feng, Bin Wang, Kun-Ling Ma, Bi-Cheng Liu

**Affiliations:** 1 Institute of Nephrology, Zhong Da Hospital, Southeast University School of Medicine, No. 87 Dingjiaqiao Rd, Nanjing, Jiangsu China; 2 Wuxi People’s Hospital Affiliated To Nanjing Medical University, Wuxi, Jiangsu China

**Keywords:** Diabetic kidney disease, Bioinformatics, Biomarker, Urinary mRNA, Microarray

## Abstract

**Background:**

Diabetic kidney disease (DKD) is the leading cause of end-stage kidney disease (ESKD) in the world. Emerging evidence has shown that urinary mRNAs may serve as early diagnostic and prognostic biomarkers of DKD. In this article, we aimed to first establish a novel bioinformatics-based methodology for analyzing the “urinary kidney-specific mRNAs” and verify their potential clinical utility in DKD.

**Methods:**

To select candidate mRNAs, a total of 127 Affymetrix microarray datasets of diabetic kidney tissues and other tissues from humans were compiled and analyzed using an integrative bioinformatics approach. Then, the urinary expression of candidate mRNAs in stage 1 study (n = 82) was verified, and the one with best performance moved on to stage 2 study (n = 80) for validation. To avoid potential detection bias, a one-step Taqman PCR assay was developed for quantification of the interested mRNA in stage 2 study. Lastly, the in situ expression of the selected mRNA was further confirmed using fluorescent in situ hybridization (FISH) assay and bioinformatics analysis.

**Results:**

Our bioinformatics analysis identified sixteen mRNAs as candidates, of which urinary *BBOX1* (uBBOX1) levels were significantly upregulated in the urine of patients with DKD. The expression of uBBOX1 was also increased in normoalbuminuric diabetes subjects, while remained unchanged in patients with urinary tract infection or bladder cancer. Besides, uBBOX1 levels correlated with glycemic control, albuminuria and urinary tubular injury marker levels. Similar results were obtained in stage 2 study. FISH assay further demonstrated that *BBOX1* mRNA was predominantly located in renal tubular epithelial cells, while its expression in podocytes and urothelium was weak. Further bioinformatics analysis also suggested that tubular *BBOX1* mRNA expression was quite stable in various types of kidney diseases.

**Conclusions:**

Our study provided a novel methodology to identify and analyze urinary kidney-specific mRNAs. uBBOX1 might serve as a promising biomarker of DKD. The performance of the selected urinary mRNAs in monitoring disease progression needs further validation.

**Electronic supplementary material:**

The online version of this article (10.1186/s12967-019-1818-2) contains supplementary material, which is available to authorized users.

## Background

Diabetic kidney disease (DKD), one of the most common complications of diabetes mellitus (DM), is the leading cause of end-stage kidney disease (ESKD) [1]. As the prognosis for patients diagnosed with DKD is not optimistic, early detection and risk classification of DKD are of paramount importance for providing appropriate and timely therapies to slow the progression to ESKD.

Currently, the microalbuminuria (MA) test is recognized as the most important diagnostic measure of DKD. However, emerging evidence has shown that MA is not an ideal diagnostic tool. A recent autopsy study demonstrated that patients with diabetes mellitus (DM) may have advanced renal pathological changes before the onset of MA [2]. Over 20% of individuals with type 1 diabetes without MA or macroalbuminuria reached stage 3 chronic kidney disease (CKD) [3]. For these reasons, novel markers which can identify patients with DKD, as well as those with a high risk of developing DKD are urgently needed.

Recently, analysis of urinary mRNAs has emerged as a novel non-invasive method for monitoring a variety of kidney diseases, including CKD [4], renal-allograft rejection [5], renal fibrosis [6] and DKD [7]. In previous studies, the candidate mRNAs were selected from molecules reported to have a role in disease development. This literature-based screening approach, however, is often biased and low-throughput. Moreover, it does not account for the interference from other urinary cells (e.g. bladder cells, inflammatory cells and cancer cells), which imposes considerable limitations when interpreting the origin of urinary mRNAs.

To address the above challenges, a novel bioinformatics-based methodology for selecting urinary mRNAs that more accurately reflect the in situ molecular changes in kidney was established in this study. Then, based on this novel approach, a two-stage study to discover potential biomarkers for DKD was further performed.

## Methods

### Overall design for the bioinformatics analysis

**Fig. 1:**
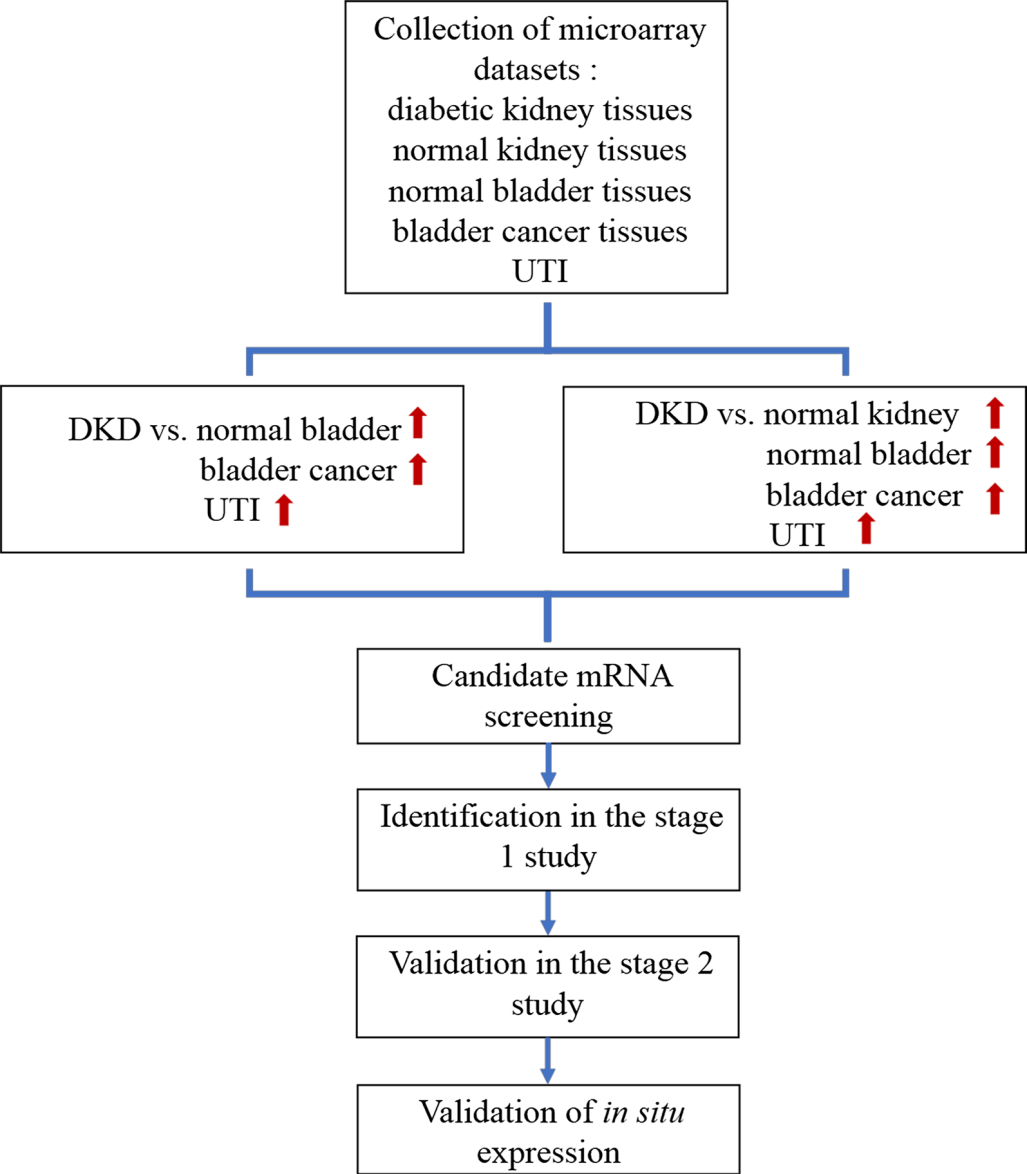
The overall study design

The overall study design is shown in Fig. 1. Microarray datasets of the following tissues were collected: (1) diabetic kidney tissues (including glomerular and tubular compartments), (2) normal kidney tissues (including glomerular and tubular compartments), (3) normal bladder tissues, (4) bladder cancer (BC) tissues, and (5) inflammatory cells from patients with urinary tract infection (UTI). Next, the transcriptome profiles of these tissues were compared and two types of differentially expressed genes (DEGs) were identified as candidate biomarkers: (1) upregulated genes with (a) highest fold changes (FCs) in diabetic kidney tissues compared with normal kidney tissues and (b) low expression in normal bladder, BC tissues and UTI-derived inflammatory cells; (2) upregulated genes with (a) highest FCs in diabetic kidney tissues compared with normal bladder tissues and (b) low expression in BC tissues and UTI-derived inflammatory cells. Next, using a targeted polymerase chain reaction (PCR) array, the urinary expression of the candidate mRNAs was determined in stage 1 study, and the one with best performance moved on to stage 2 study for validation. To avoid potential detection bias, a one-step PCR assay based on Taqman probes was developed for quantification of the interested mRNA in stage 2 study. Lastly, the in situ expression of the interested mRNA was determined by fluorescent in situ hybridization (FISH) assay and bioinformatics analysis.

### Datasets collection

The gene expression datasets used in this study were compiled using the gene expression omnibus (GEO; https://www.ncbi.nlm.nih.gov/geo/). For datasets of normal human glomeruli, tubules and bladder tissues, the primary search criteria were set to “glomerulus OR glomeruli”, “tubulus OR tubules” and “bladder” respectively. For DKD, the primary search criteria were set to “diabetic nephropathy OR diabetic kidney disease OR DKD”. For UTI-derived inflammatory cells and BC tissues, the primary search criteria were set to “urinary tract infection OR UTI” and “bladder AND (cancer OR tumor)”, respectively. To minimize platform variations, the platform filtering criterion was set to “Affymetrix U133″, which included three types of microarrays (Human Genome U133 Plus 2.0 Array, Human Genome U133A 2.0 Array, and Human Genome U133A&B). The searching criteria for other types of CKD can be found in our previous study [8].

### Processing microarray datasets

Data processing was performed using Bioconductor (version 3.4) in R software (version × 64 3.3.2) according to the following steps [9]:

1. Before included in the analysis, the quality of each dataset was examined using the general quality control (QC) stats in the simpleaffy package and RNA degradation analysis in the affy package. Those unqualified datasets were discarded.
2. For analysis involving different types of microarrays, the 22,215 Affymetrix identifiers shared by both the Human Genome U133 Plus 2.0 Array and Human Genome U133A Array were extracted. Datasets of the same category were pooled to expand the sample size. Then the relative log expression graph was used to evaluate the consistency among datasets, and those with significant bias were discarded.
3. The robust multi-array average method in the affy package was used to preprocess the original data. Then the principal component analysis was used to detect potential batch effects of pooled datasets. Lastly, a linear model was fit to the normalized data to obtain an expression measure for each probe set on qualified datasets.
4. The empirical Bayes method was used to select DEGs for each disease. Statistically significant DEGs were defined as those with p-values < 0.05 after adjustment by the Benjamini–Hochberg method and FCs > 2.

### Study population

This 2-stage cross-sectional study was approved by the Ethical Committee of Zhong Da Hospital of Southeast University. All participants provided written informed consent. In the stage 1 study, four categories of participants (n = 82) were enrolled: (1) normoalbuminuric patients with DM (NA group: albumin-creatine ratio [ACR] < 30 mg/g); (2) patients with DKD; (3) patients with UTI (UTI group) or BC (BC group); and (4) healthy controls (HCs). Patients with DKD were further divided into three subgroups: (1) those with MA (MA group: ACR 30–300 mg/g); (2) those with overt albuminuria (OA group: ACR > 300 mg/g); and (3) those with ESKD (ESKD group). In the stage-2 study, another 80 participants were enrolled (NA = 20; MA = 20; OA = 20; HC = 20).

DKD was diagnosed according to the Kidney Disease Outcomes Quality Initiative (KDOQI) guidelines of 2007 (10). The inclusion criteria were set as follows: at least 5 years from diagnosis of type 2 diabetes, the presence of diabetic retinopathy, and an elevated ACR. The exclusion criteria were as follows: infection, signs or symptoms of other systemic diseases, or suspected non-diabetic kidney disease. ESKD was defined as the onset of dialysis or estimated glomerular filtration rate (eGFR) < 15 ml/min/1.73 m^2^. The eGFR was calculated according to modified modification of diet in renal disease (MDRD) equations [10].

The HCs were enrolled from the Zhong Da Hospital Health Care Center, all of whom met the following criteria: (1) no record of abnormal renal function (eGFR < 90 mL/min/1.73 m^2^); (2) normal routine urinalysis, ACR, and 24 h urinary protein test results; (3) no record of hypertension, diabetes, hyperlipidaemia, or hyperuricaemia; and (4) no family history of kidney diseases. Moreover, all patients with UTI and BC have no evidence of kidney diseases or diabetes.

### PCR assay

In the stage-1 study, a targeted PCR array was fabricated to detect differentially expressed mRNAs with highest FCs. The primer sets were designed using PRIMER 5 software according to optimized experimental conditions. Two housekeeping genes (*B2M* and *RPL27*) were used to normalize data. Positive PCR control (PPC) and reverse transcription control (RTC) were set into the array to monitor PCR reaction performance. Cycling conditions were set as follows: 95 °C for 10 min, followed by 40 cycles of 15 s at 95 °C and 60 °C for 1 min.

In the stage-2 study, a Taqman one-step PCR assay was developed for quantification of the BBOX1 mRNA. *B2M* was used as the housekeeping gene. Cycling conditions were set as follows: 95 °C for 10 min, followed by 45 cycles of 20 s at 95 °C and 60 °C for 45 s. All primer sequences can be found in the additional files (Additional file 1: Table S1). All PCR assays were performed using an ABI PRISM7700 system (Applied Biosystems).

In order to further test the reproducibility and sensitivity of the Taqman PCR assay, the cycle threshold (Ct) values of BBOX1 and B2M in different amounts of total urinary RNA (500 ng, 50 ng, 5 ng and 0.5 ng) were measured for three times. The reproducibility was measured by the coefficient of variation (CV) according to the following formula $c_{v} = \frac{\sigma }{\mu }$, where σ and μ stand for the standard deviation(SD) and mean of repeated measurements, respectively.

### Urinary mRNA samples collection and measurement

First morning urine samples were collected and centrifuged at 3000*g* for 30 min at 4 °C within 2 h of collection to obtain the urinary sediments. The sediments were then resuspended in 1.5 ml DEPC-treated PBS and centrifuged at 12,000*g* for 5 min at 4 °C. RNAiso Plus (Takara) was added to preserve total RNA, and the samples were stored at − 80 °C until use. Total RNA was extracted according to the manufacturer’s protocol (Invitrogen). Then, RNA concentrations were measured using a NanoDrop 2000 (Thermo) based on the relative absorbance ratio at 260/280 nm. The qualified RNA samples were then reverse transcribed to cDNA according to the manufacturer’s protocol (Takara), which were stored at − 20 °C until use.

### Confirmation of in situ mRNA expression

The Ethical Committee of Zhong Da Hospital of Southeast University approved the use of human samples for the experiments outlined in this study. FISH assay was performed on 2-μm-thick sections of diabetic kidney tissues and normal urothelium to determine the in situ mRNA expression levels. Additionally, kidney tissues were co-stained with podocalyxin antibody to detect the expression of *BBOX1* mRNA in podocytes.

Briefly, sections were first deparaffinized and dehydrated in dimethylbenzene and ethanol, followed by rinsing twice in distilled water for 5 min each. After pre-treatment with pepsin and permeabilization, the sections were treated with a *BBOX1* gene probe mix (Exiqon, sequence:5′-AGTAA TCCAC TCCAA TGTCT GT-3′) overnight at room temperature. To stain podocytes, the slides were additionally incubated with labeled anti-human podocalyxin monoclonal antibodies(Abcam) at a dilution of 1:100 overnight. Nuclei were counterstained with 4′,6-diamidino-2-phenylindole (DAPI) and coverslips were fixed with nail polish. Analysis of fluorescence signals was performed using a Nikon Eclipse C1 epifluorescence microscope with interference filters (AHF Analysentechnik AG).

### Statistical analysis

R software (version ×64 3.3.2) and Graphpad Prism 7.0 were used for all other statistical analysis and figure construction, respectively. The Shapiro–Wilk test was used to determine the normality of the data. Numeric results with a normal distribution were presented as the mean ± SD. Non-normal numeric results were presented with the interquartile range (IQR). Analysis of variance (ANOVA) was applied to compare the means of normalized data. For skewed data, the Kruskal–Wallis test was applied. The frequencies were compared using the Chi squared test. The correlation between gene expression levels and clinical parameters were analyzed using Spearman’s rank-order test. The discriminative power of the biomarker was evaluated by generating receiver operating characteristic (ROC) curves. The area under the curve (AUC) was used to assess the overall discriminatory power. An AUC of 0.6–0.7 was considered poor, 0.7–0.8 was considered moderate, 0.8–0.9 was considered good, and > 0.9 was considered excellent. Optimal cut-offs were determined by selecting the data points that maximized the sum of specificity and sensitivity on the ROC curve. Two-tailed p < 0.05 was considered statistically significant.

## Results

### Characteristics of microarray datasets

After screening and quality control tests, a total of 127 qualified datasets (DKD glomeruli: n = 7, normal glomeruli: n = 40, DKD tubules: n = 11, normal tubules: n = 22, normal bladder: n = 23, UTI: n = 5, BC: n = 19) were included for candidate screening (Table 1). These datasets were obtained from GSE37463 [11], GSE24152(unpublished study), GSE47185 [12], GSE35489 [13], GSE7476 [14], GSE11783 [15], GSE18810(unpublished study), GSE21785 [16] and GSE20602 [17]. All datasets were generated by either the Human Genome U133 Plus 2.0 Array or Human Genome U133A Array. Of note, all subjects with DKD had biopsy-proven diabetic nephropathy. Normal renal tissues were obtained from either pre-transplant living donors or patients after tumor nephrectomy.

**Table 1:** Included microarray datasets after quality control

Microarray datasets	Number	Resources	Platforms
DKD glomeruli	7	GSE47185	U133 Plus 2.0
Normal glomeruli	40	GSE37460 GSE47185 GSE20602 GSE21785	U133 Plus 2.0 U133A
DKD tubules	11	GSE47185	U133 Plus 2.0
Normal tubules	22	GSE47185 GSE35487	U133 Plus 2.0 U133A
Normal bladder	23	GSE7476 GSE11783	U133 Plus 2.0
Polymorphonuclear leukocytes (UTI)	5	GSE18810	U133 Plus 2.0
Bladder cancer	19	GSE7476 GSE24152	U133 Plus 2.0

### Identification of candidate mRNAs

**Fig. 2:**
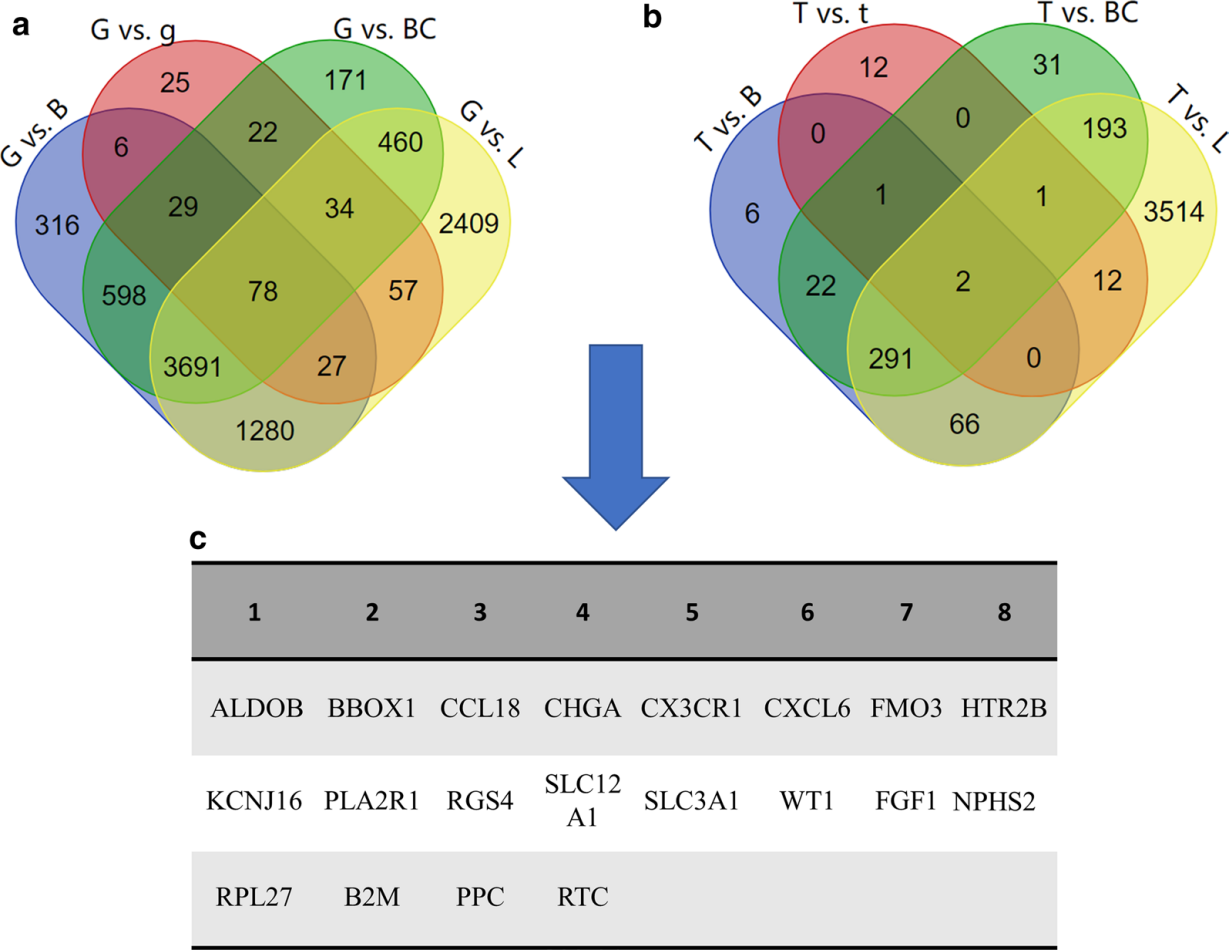
The identification of candidate mRNAs. **a** The Venn diagram of DEGs identified in glomerular compartments of diabetic kidney tissues; **b** The Venn diagram of DEGs identified in tubular compartments of diabetic kidney tissues; **c** A display of selected candidate genes in the PCR array. G, glomerular compartments of diabetic kidney tissues; T, tubular compartments of diabetic kidney tissues; g, glomerular compartments of normal kidney tissues; t, tubular compartments of normal kidney tissues; B, normal bladder tissues; BC, bladder cancer tissues; L, polymorphonuclear leukocytes in urinary tract infection

As shown in Fig. 2, after comparing the gene expression patterns in glomeruli from diabetic kidney tissues and others, a total of 9203 upregulated DEGs were identified (DKD glomeruli vs. normal bladder, n = 6025; DKD glomeruli vs. normal glomeruli, n = 278; DKD glomeruli vs. BC, n = 5083; DKD glomeruli vs. UTI-derived polymorphonuclear leukocytes, n = 8036), of which seventy-eight were co-differentially expressed.

For tubules from diabetic kidney tissues, a total of 4151 upregulated DEGs were identified (DKD tubules vs. normal bladder, n = 388; DKD tubules vs. normal tubules, n = 28; DKD tubules vs. BC, n = 541; DKD tubules vs. UTI-derived polymorphonuclear leukocytes, n = 4079), of which two were co-differentially expressed.

According to the rules described in the Methods section (Overall design for the bioinformatics analysis), sixteen mRNAs were finally selected as candidate biomarkers. Detailed lists of differentially expressed genes are shown in the additional files (Additional file 2: Table S2, Additional file 3: Table S3, Additional file 4: Table S4, Additional file 5: Table S5, Additional file 6: Table S6, Additional file 7: Table S7, Additional file 8: Table S8, Additional file 9: Table S9).

### Characteristics of the study population

A total of 82 participants were enrolled in stage 1 study (HC: n = 14; NA: n = 16; MA: n = 11; OA; n = 12, ESKD: n = 13; UTI: n = 8; BC: n = 8). Table 2 shows the demographic and clinical characteristics of included participants. The mean ages of participants were similar in the HC, NA and UTI group, but those in the MA, NA, ESKD and BC group were older. In addition, the renal function of patients in the MA, OA and ESKD group was significantly poorer than those of HCs. Patients in the UTI and BC group all had normal albuminuria, blood glucose levels and eGFR.

**Table 2:** Basic characteristics of participants in stage-1 study

	HC	NA	MA	OA	ESKD	UTI	BC
Number	14	16	11	12	13	8	8
Age	47.0 ± 8.6	49.9 ± 10.2	64.3 ± 6.7*	60.4 ± 8.9*	64.3 ± 12.2*	49.4 ± 13.3	62.3 ± 10.1*
Gender (male/female)	9/5	7/9	4/7	10/2	11/2	5/3	2/6
eGFR (ml/min/1.73 m^2^)	101.4 [95.9–110.3]	107.6 [90.6–136.3]	71.1 [40.3–112.1]*	50.5 [30.7–80.7]*	11.9 [8.6–14.7]*	100.4 [84.2–111.9]	113.8 [93.9–140.9]
ACR (mg/g)	5.2 [2.9–8.5]	6.4 [4.8–12.6]	120.6 [85.8–216.6]*	685.9 [414.4–3094.0]*	643.5 [546.3–890.3]*	8.0 [4.4–12.7]	10.7 [7.3–15.0]
Glu (mmol/L)	5.7 [5.2–5.9]	12.0 [8.4–16.8]*	8.9 [6.8–16.4]*	9.6 [6.4–12.8]*	5.7 [4.8–9.2]	5.8 [4.9–6.0]	5.7 [5.5–5.9]
HbA1c (%)	4.9 [4.6–5.3]	10.1 [7.8–10.6]*	8.3 [6.8–11.2]*	8.6 [6.2–9.0]*	6.1 [5.3–8.0]	5.3 [4.9–5.5]	4.9 [4.6–5.5]
SBP (mmHg)	119.2 ± 7.5	126.3 ± 9.5	137.5 ± 13.1*	147.6 ± 21.9*	155.5 ± 24.6*	121.9 ± 11.5	127.1 ± 12.4
DBP (mmHg)	76.4 ± 6.6	79.3 ± 8.2	81.6 ± 9.1	81.7 ± 9.8	78.3 ± 12.6	74.9 ± 5.5	76.4 ± 6.5
Urinary NAG/Cr (U/mol)	0.43 [0.38–0.72]	0.60 [0.44–1.14]	1.30 [0.45–3.70]*	1.90 [0.84–4.81]*	1.71 [1.14–2.60]*	0.51 [0.37–0.74]	1.33 [0.54–2.34]*

Glu, blood glucose; SBP, systolic blood pressure; DBP, diastolic blood pressure

* p < 0.05 compared with HCs

In the stage-2 study, another 80 participants were enrolled (NA: n = 20; MA: n = 20; OA: n = 20; HC: n = 20). As shown in Table 3, there was no significant difference in age among different groups. Patients in the OA group, but not the NA or MA group, had lower eGFR than HCs.

**Table 3:** Basic characteristics of participants in stage-2 study

	HC	NA	MA	OA
Number	20	20	20	20
Age	49.7 ± 7.5	50.6 ± 15.3	50.1 ± 13.7	55.7 ± 14.0
Gender (male/female)	13/7	11/9	13/7	14/6
eGFR (ml/min/1.73 m^2^)	102.6 [95.0–114.6]	102.6 [77.1–115.0]	88.5 [79.2–138.8]	82.9 [32.8–123.8]*
ACR (mg/g)	10.1 [4.7–15.1]	12.0 [5.9–20.5]	78.4 [58.8–149.3]*	1503.0 [619.9–3743.0]*
Glu (mmol/L)	4.9 [4.3–5.2]	8.6 [7.0–11.1]*	10.1 [7.5–12.7]*	12.3 [7.5–15.0]*
HbA1c (%)	4.9 [4.7–5.2]	8.3 [7.0–10.2]*	8.5 [6.8–9.3]*	9.1 [8.2–11.1]*
SBP (mmHg)	116.5 ± 9.5	123.2 ± 10.6	135.4 ± 12.3*	145.5 ± 18.6*
DBP (mmHg)	74.3 ± 5.8	80.2 ± 7.3	82.5 ± 10.1*	85.5 ± 7.8*
Urinary NAG/Cr (U/mol)	0.48 ± 0.14	0.69 ± 0.27	1.17 ± 0.44*	1.93 ± 0.53*

* *p *< 0.05 compared with HCs

### Urinary BBOX1 mRNA (uBBOX1) expression was upregulated in patients with DKD

**Fig. 3:**
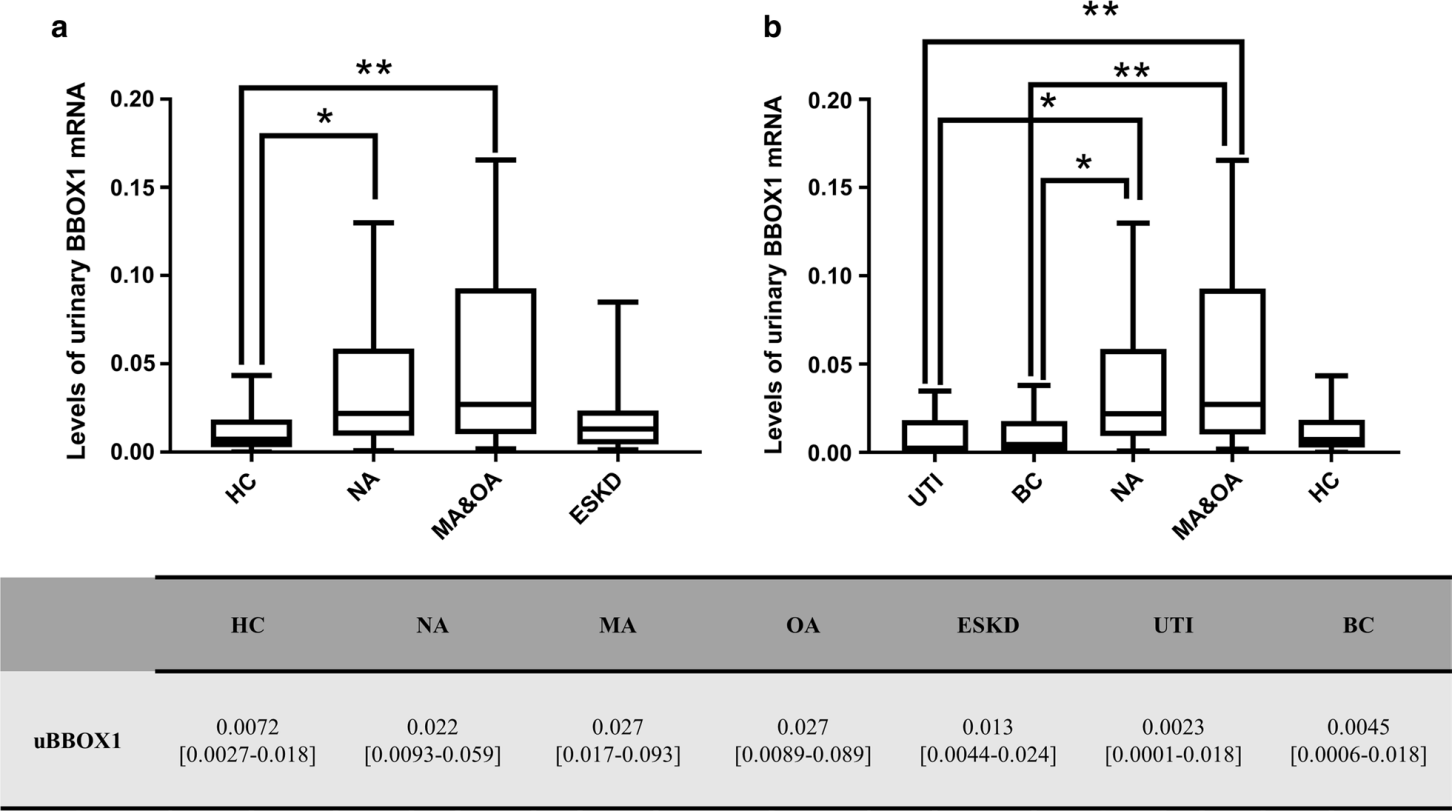
The differential expression of uBBOX1 among different populations in stage 1 study. **a** uBBOX1 levels were significantly elevated in the NA, MA and OA group, but not the ESKD group; **b** uBBOX1 levels of the UTI and BC group were significantly lower than those of the NA, MA and OA group. *p < 0.05; **p < 0.01

To verify the differential expression of candidate mRNAs in the urine, the urinary mRNA profile of each participant of stage 1 study was measured using the self-assembly PCR array. As a result, four urinary mRNAs (*BBOX1*, *CCL18*, *NPHS2*, and *SLC3A1*) were found to be upregulated in the patients with DKD (MA and OA) compared to HCs (Table 4). After adjusting for false positives using the Benjamini–Hochberg method, uBBOX1 remained significantly different (adjusted *p *= 0.036). Next, its expression among different groups was further examined in stage 1 study. The median relative expression of uBBOX1 was 0.0072 in the HC group (IQR 0.0072 [0.0027–0.018]). Its expression was increased threefold in the NA group (IQR 0.022 [0.0093–0.059], adjusted *p *= 0.036) and 3.1-fold in the non-ESKD DKD groups (IQR 0.027 [0.010–0.093], adjusted *p *= 0.0083), respectively (Fig. 3). Interestingly, its expression was not significantly changed in patients with ESKD compared to the HCs (IQR 0.013 [0.0044–0.024]). However, patients in the NA, MA, and OA group all had similar uBBOX1 expression. In addition, uBBOX1 levels of UTI or BC group were not elevated in comparison with those of HCs, and were significantly lower than those of the NA group (UTI vs. NA: adjusted *p* = 0.012; BC vs. NA: adjusted *p* = 0.025) and the non-ESKD DKD groups (UTI vs. MA and OA: adjusted *p* = 0.0047; BC vs. MA and OA: adjusted *p* = 0.0086).

**Table 4:** A list of differentially expressed urinary mRNAs

	Controls	DKD (MA and OA)	Fold changes of the median	p values	Adjusted p values
BBOX1	0.0072 [0.0027–0.018]	0.027 [0.010–0.093]	3.1	0.0021	0.036
CCL18	0.0017 [0.00025–0.0061]	0.016 [0.0019–0.039]	9.4	0.0094	0.078
NPHS2	0.039 [0.0069–0.088]	0.085 [0.026–0.35]	2.2	0.024	0.136
SLC3A1	0.0023 [0.00083–0.0078]	0.014 [0.0018–0.028]	6.1	0.031	0.132

Adjusted p values were calculated by the Benjamini–Hochberg method

ns, not significant

### Correlation between uBBOX1 expression and clinical parameters

**Fig. 4:**
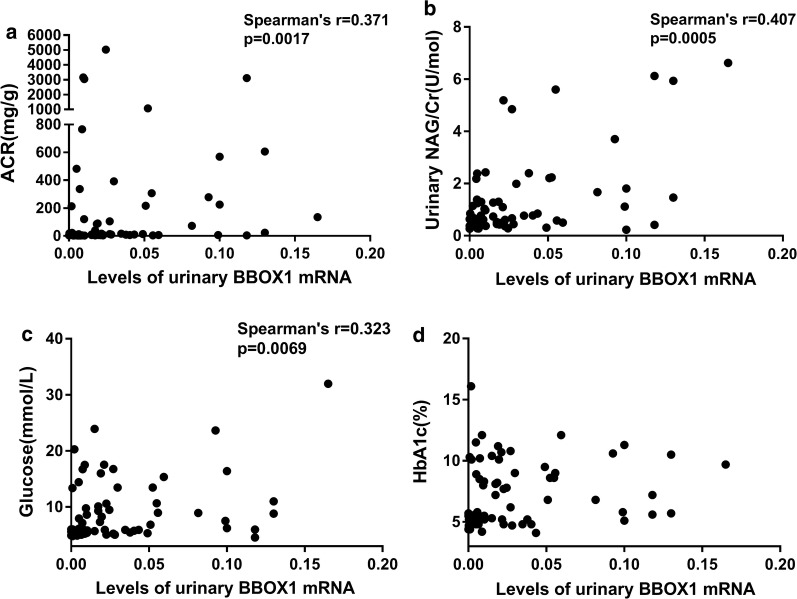
Correlation between uBBOX1 expression and clinical parameters (patients with ESKD not included). uBBOX1 positively correlated with the levels of urinary ACR (**a**), urinary NAG (**b**), blood glucose (**c**) and HbA1c (**d**)

The correlation between uBBOX1 expression and clinical parameters was further analyzed with patients with ESKD excluded. As shown in Fig. 4, the levels of uBBOX1 were positively correlated with albuminuria (Spearman’s r = 0.371, *p *= 0.0017), urinary *N*-acetyl-beta-d-glucosaminidase (NAG) levels (Spearman’s r = 0.407, *p *= 0.0005), blood glucose levels (Spearman’s r = 0.323, *p *= 0.0069) and HbA1c levels (Spearman’s r = 0.292, *p *= 0.015). However, significant correlation between uBBOX1 and blood pressure or eGFR was not found.

### The discriminative performance of uBBOX1

**Fig. 5:**
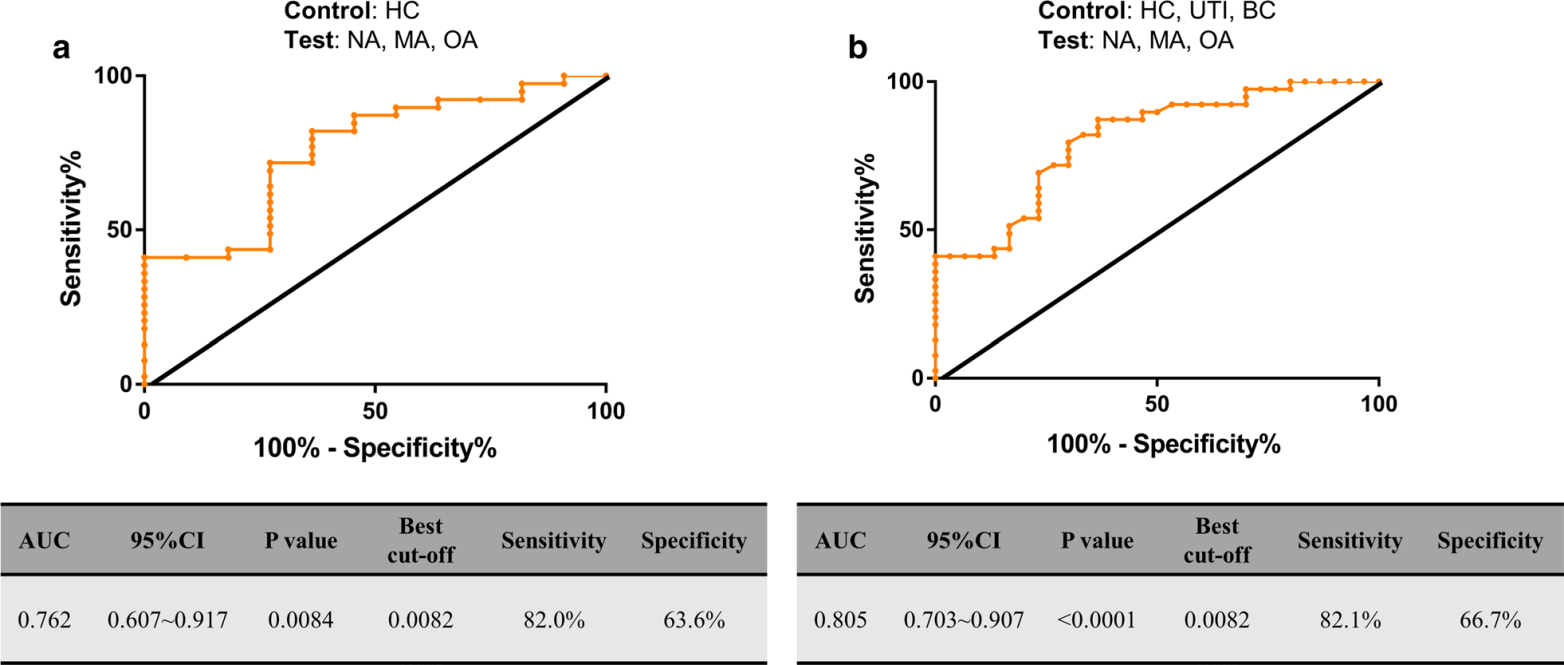
The discriminative performance of uBBOX1. **a** uBBOX1 yielded an AUC of 0.762 in discriminating the test group (NA, OA and MA) from HC group; **b** uBBOX1 yielded an AUC of 0.805 in discriminating the test group (NA, OA and MA) from non-diabetes groups (HC, UTI and BC). AUC, area under the curve; 95% CI, 95% confidence interval

Next, ROC analysis was performed to further test the overall accuracy of uBBOX1 in discriminating different groups of participants enrolled in the stage 1 study. As shown in Fig. 5, with HCs as the control group and patients with diabetes and non-ESKD DKD as the test group, uBBOX1 had an AUC of 0.762 (*p *= 0.0084). At its optimal cut-off value of 0.0082, uBBOX1 yielded a specificity of 63.6% and a sensitivity of 82.0%. When patients with UTI and BC were added to the control group, the AUC for uBBOX1 changed to 0.805 (*p *< 0.0001). Interestingly, the optimal cut-off value remained at 0.0082, where uBBOX1 yielded a specificity of 66.7% and a sensitivity of 82.1%.

### Performance validation of uBBOX1 in stage 2 study

To avoid potential detection bias, the reproducibility and sensitivity of the one-step Taqman PCR assay were tested. As shown in Table 5, both BBOX1 and B2M mRNAs can be detected in the reaction systems containing as low as 0.5 ng total urinary RNA/10ul with low variance (average CVs for Ct values of BBOX1: 0.26%; average CVs for Ct values of B2M: 0.14%).

**Table 5:** Reproducibility and sensitivity of the one-step Taqman PCR assay

	CT1	CT2	CT3	CV (%)
500 ng total RNA/10 µl				
BBOX1	25.29	25.54	25.38	0.49
B2M	15.51	15.58	15.52	0.24
50 ng total RNA/10 µl				
BBOX1	28.19	28.26	28.24	0.10
B2M	18.84	18.81	18.85	0.09
5 ng total RNA/10 µl				
BBOX1	31.55	31.43	31.42	0.19
B2M	22.25	22.29	22.2	0.17
0.5 ng total RNA/10 µl				
BBOX1	33.87	33.86	34.06	0.27
B2M	25.74	25.73	25.71	0.05

**Fig. 6:**
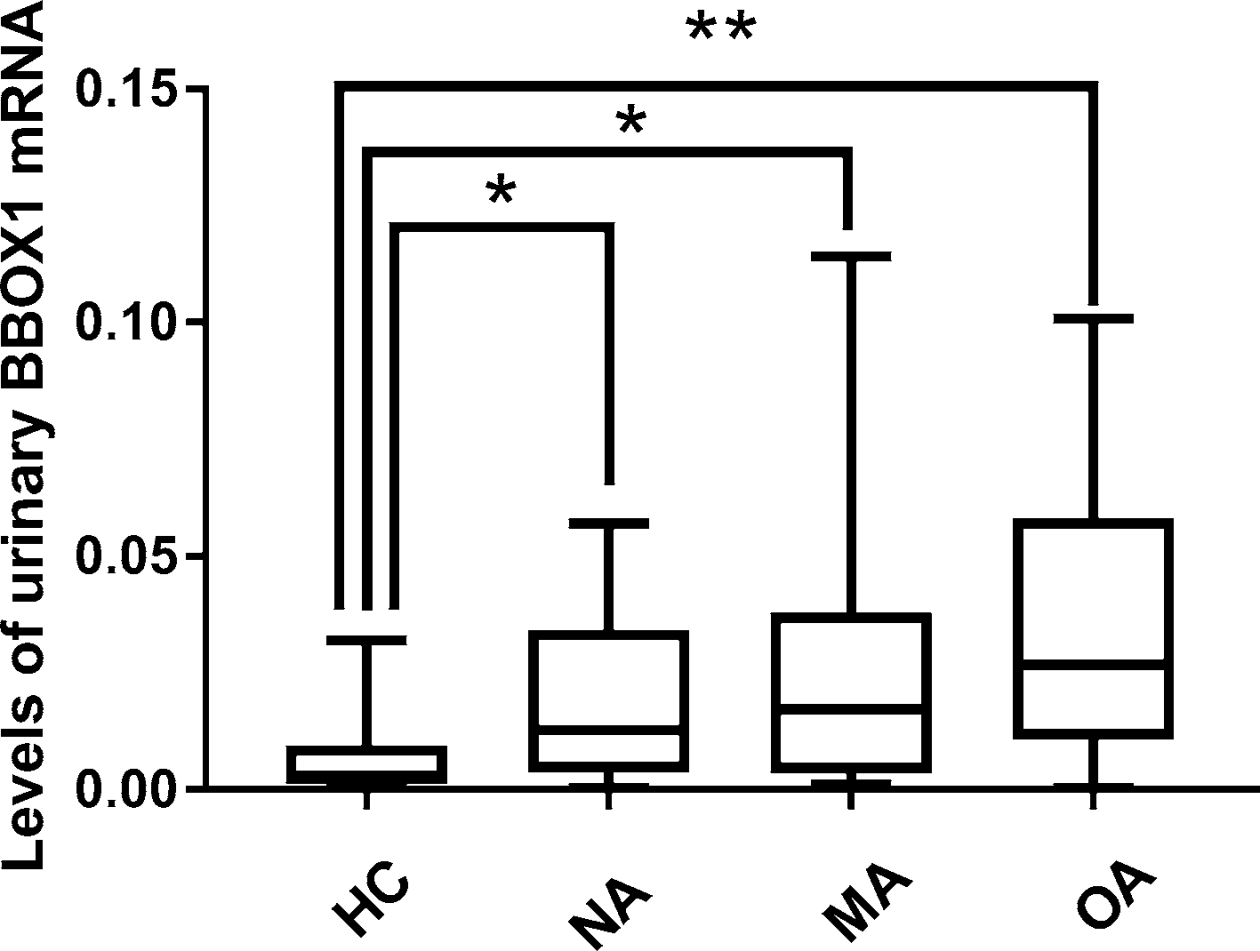
The differential expression of uBBOX1 among different populations in stage 2 study. uBBOX1 levels were significantly elevated in NA, MA and OA group, *p < 0.05; **p < 0.01

**Fig. 7:**
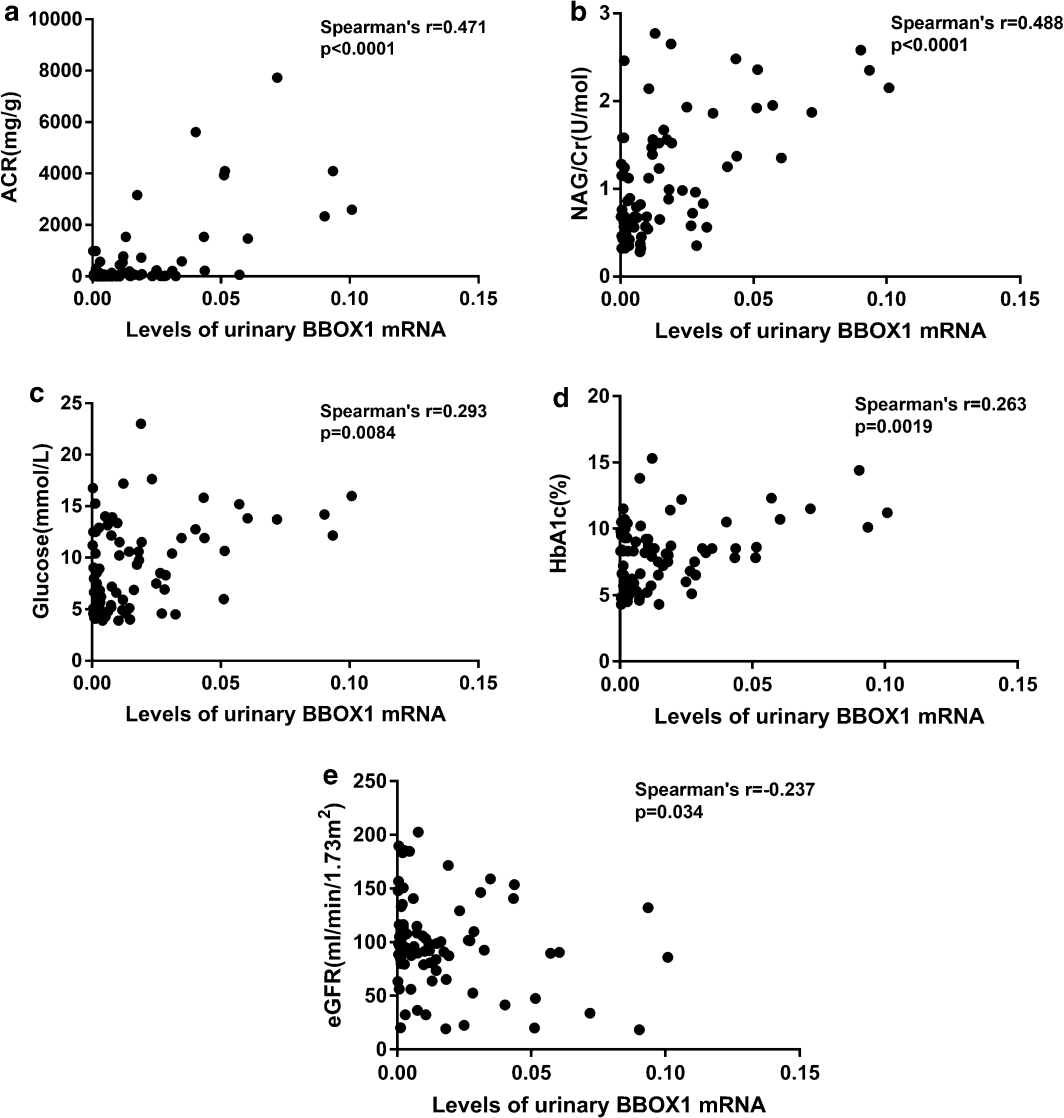
Correlation between uBBOX1 expression and clinical parameters (stage 2 study). uBBOX1 positively correlated with the levels of urinary ACR (**a**), urinary NAG (**b**), blood glucose (**c**) and HbA1c (**d**), while negatively correlated with the levels of eGFR (**e**)

Then using this one-step Taqman PCR assay, the uBBOX1 levels of all participants in study 2 were measured. As shown in Fig. 6, the expression of uBBOX1 was significantly upregulated in patients of NA (IQR 0.0064 [0.0018–0.017], adjusted *p* = 0.0441), MA (IQR 0.0086 [0.0018–0.019], adjusted *p* = 0.0205) and OA group (IQR 0.027 [0.011–0.058], adjusted *p* = 0.0013) compared with HCs (IQR 0.0030 [0.0014–0.0074]). However, significant difference of uBBOX1 levels within the NA, MA and OA group was not found. In correlation analysis (Fig. 7), uBBOX1 also positively correlated with urinary ACR levels (Spearman’s r = 0.471, *p *< 0.0001), urinary NAG levels (Spearman’s r = 0.488, *p *< 0.0001), blood glucose levels (Spearman’s r = 0.293, *p *= 0.0084), HbA1c levels (Spearman’s r = 0.263, *p *= 0.019). A weak negative correlation between eGFR and uBBOX1 was also found (Spearman’s r = 0.263, *p *= 0.019).

### *BBOX1* mRNA in situ expression in diabetic kidney tissues

**Fig. 8:**
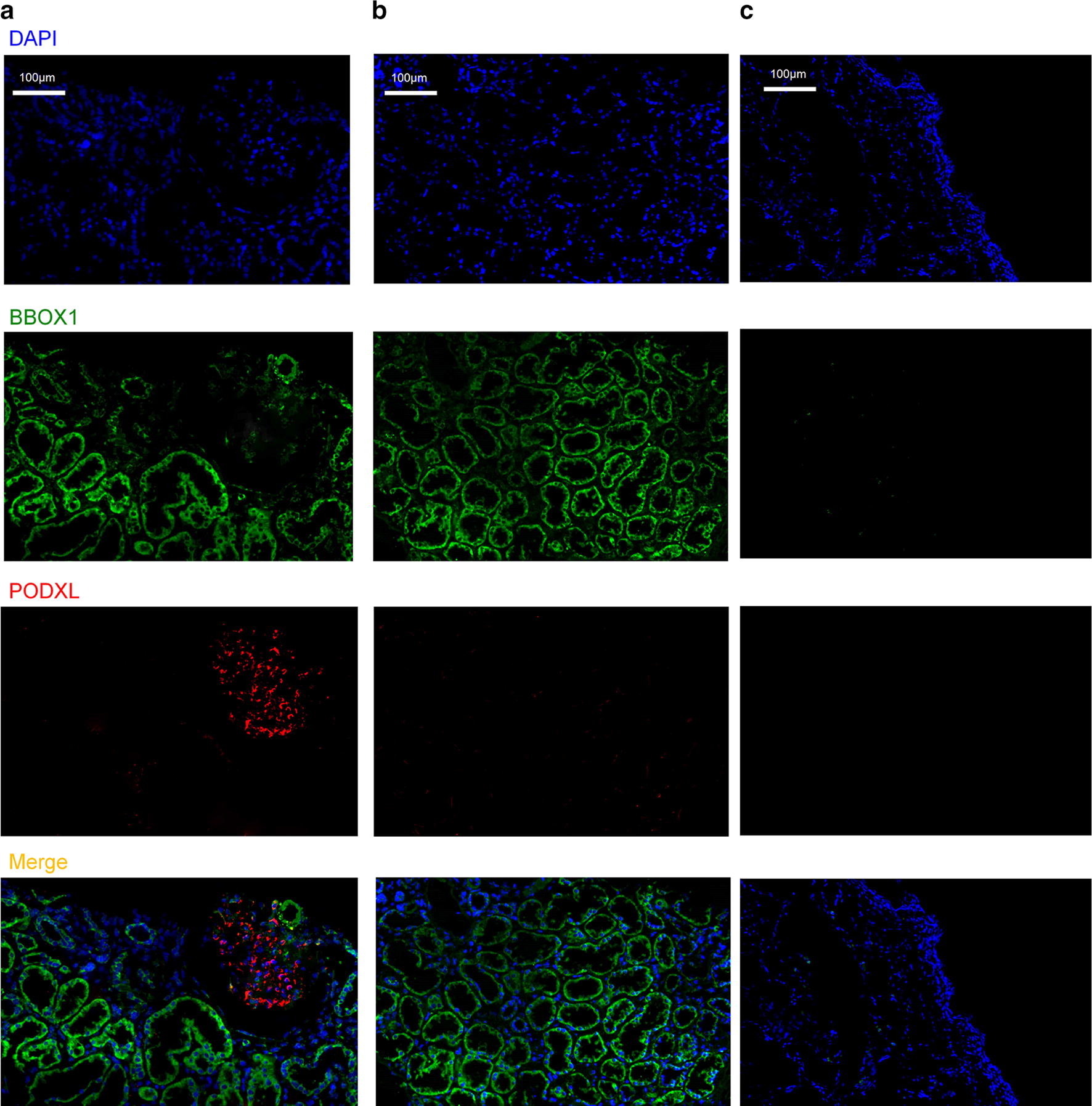
In situ expression of *BBOX1* mRNA in diabetic kidney tissue and normal bladder tissue. *BBOX1* mRNA expression in the glomerular compartment of diabetic kidney tissue (**a**), the tubular compartment of diabetic kidney tissue (**b**) and the normal bladder tissue (**c**). DAPI, 4′,6-diamidino-2-phenylindole; PODXL, podocalyxin

Next, the in situ expression of *BBOX1* mRNA in human tissues was examined. As shown in Fig. 8, *BBOX1* mRNA was highly expressed in the tubular compartments of diabetic kidney tissues, while its expression in glomerular compartments including podocytes was weak. Moreover, *BBOX1* mRNA was poorly expressed in normal bladder tissues, indicating that detached tubular epithelial cells (TECs) primarily contributed to the elevated uBBOX1 levels. Next, we asked whether the tubular expression of *BBOX1* mRNA would change in the context of DKD. As shown in Table 6, bioinformatics analysis involving 33 microarray datasets showed that tubular expression of *BBOX1* mRNA was not significantly changed in diabetic kidney tissues (FC: 0.745, adjusted *p *= 0.136).

**Table 6:** Relative expression of *BBOX1* mRNA in various types of chronic kidney disease compared with normal kidney tissues

Disease	Compartment	Relative expression	Adjusted p values
DKD	Tubules	0.745	0.136
HN	Tubules	0.929	0.438
FSGS	Tubules	1.17	0.134
IgAN	Tubules	0.965	0.662
MN	Tubules	1.28	0.004

HN, hypertensive nephropathy; FSGS, focal segmental glomerulosclerosis; IgAN, IgA nephropathy; MN, membranous nephropathy

### The differential expression of BBOX1 mRNA in other types of CKD

To expand the potential use of uBBOX1, the differential expression of *BBOX1* mRNA in other types of CKD was further examined using an integrative bioinformatics approach. The basic characteristics of the included datasets have been described in our previous study [8]. As shown in Table 6, compared with normal kidney tissues, tubular *BBOX1* mRNA expression was either not significantly changed or slightly upregulated in other types of CKD including hypertensive nephropathy (relative expression: 0.929, adjusted *p *= 0.438), focal segmental glomerulosclerosis (relative expression: 1.17, adjusted *p *= 0.134), IgA nephropathy (relative expression: 0.965, adjusted *p *= 0.662) and membranous nephropathy (relative expression: 1.28, adjusted *p *= 0.0045).

## Discussion

Although MA is the most common diagnostic measure for DKD, recent studies have suggested that it is suboptimal in many cases. Therefore, there is an urgent need to identify novel biomarkers to increase the accuracy of diagnostic tools.

Previous studies have applied a literature-based approach to screen candidate urinary mRNA markers for kidney diseases. This approach is inherently biased and lacks efficiency. In recent years, bioinformatics has emerged as a powerful tool for the high-throughput identification of potential biomarkers [18]. However, owing to the tremendous amount of data generated by high-throughput technology, bioinformatics analysis based on a few samples may be at a high risk of false positive results. In the present study, we developed a novel bioinformatics workflow based on over one hundred microarray datasets and found that uBBOX1, a TEC-specific mRNA, could be used as a potential biomarker of DKD.

The *BBOX1* gene (also known as BBH) is located on chromosome 11 and encodes gamma-butyrobetaine hydroxylase 1, which catalyzes the formation of L-carnitine from gamma-butyrobetaine [19]. L-carnitine deficiency is associated with skeletal myopathies, poorer renal and cardiac function, and anemia in the context of CKD [20, 21]. It also exerts antioxidant and anti-inflammatory effects on TECs in various kidney injury models [22, 23]. The kidney, the liver and the brain are three major organs where carnitine biosynthesis is highly activated [19]. Using quantitative PCR, Rigault et al. found that *BBOX1* mRNA is highly expressed in the kidney, with an increase in expression of over threefold and ninefold compared to the liver and brain, respectively [19]. Our result further demonstrated that *BBOX1* mRNA is predominantly located in TECs, while its expression in podocytes and urothelium is weak. Therefore, detached TECs and fragments are the major origin of uBBOX1. In addition, *BBOX1* mRNA expression, as revealed by our bioinformatics analysis, was not significantly changed in TECs. Hence, detecting uBBOX levels provides a novel and fast method for measuring the extent of TEC loss in the urine.

Previous studies have shown that urinary podocyte mRNAs, which reflect the extent of podocyturia, can be useful as a non-invasive marker for DKD [24, 25]. In a cohort of 1143 patients, urinary podocyte mRNAs were markedly increased in patients with glomerular diseases including DKD and even higher in progressors [26]. Recently, tubulopathy is becoming increasingly recognized as a key culprit of DKD—not only during late stages, but also at the onset of disease [27]. As a consequence of tubular injury induced by DM-related pathophysiological disturbances including albuminuria and hyperglycemia, elevated TEC excretion can be found in the urine of patients with DM [28–30]. Hyperglycemia can trigger oxidative stress and lead to aggravated injury of TECs even in non-diabetic rats [31]. Consistent with these studies, we observed that uBBOX1 levels were augmented in normoalbuminuric patients with diabetes and patients with DKD, and positively correlated with albuminuria, hyperglycemia and urinary NAG excretion, indicating its potential diagnostic value for DKD. Its correlation with eGFR, however, was relatively weak. Besides, the differences of uBBOX1 levels among the NA, MA and OA group did not reach statistical significance. Aside from the fact that the relative small sample size limits the statistical power, the sharp increase of urinary mRNA upon kidney damage may also contribute to the insignificance [32]. Another interesting phenomenon is that patients with ESKD had a decreased level of uBBOX1 compared with those in earlier stages, which may have been owing to the lack of kidney intrinsic cells in fibrotic kidneys. Taken together, these data suggest that uBBOX1 may act as an early biomarker for DKD.

To expand the potential use of uBBOX1, we also analyzed its in situ expression in other types of CKD by an integrative bioinformatics method. We found that tubular *BBOX1* mRNA expression is quite stable in hypertensive nephropathy and various types of glomerulonephritis, suggesting that elevated uBBOX1 may also act as an indicator of tubular injury in these kidney diseases. Further studies are still required to verify this hypothesis.

Other strengths of this study include: (1) we developed a convenient and effective detection method based on Taqman probes for large-scale clinical verification and transformation; (2) we pre-planned to minimize the potential interference from urinary cells in the context of UTI and BC. Unsurprisingly, uBBOX1 expression was not substantially increased in patients with UTI or BC, supporting the efficacy of this top-down study design. Notably, uBBOX1 was found to be highly effective in discriminating patients with diabetes from HCs and those with confounding diseases.

Some limitations of our study should be considered. Firstly, the study design was cross-sectional with a relatively small sample size, which reduced the significance of the study. Additionally, the diagnosis of DKD in our study was based on laboratory parameters rather than pathological parameters. Large-scale prospective studies are still needed to further validate the prognostic role of uBBOX1 for DKD and other CKDs.

## Conclusions

In conclusion, our study provided a novel methodology to identify and analyze urinary kidney-specific mRNAs. Urinary BBOX1 mRNA might serve as a promising biomarker of early detection of DKD. The performance of the selected urinary mRNAs in monitoring DKD progression needs further validation.

## Additional files

**Additional file 1: Table S1.** Primer sequences of candidate mRNAs.

**Additional file 2: Table S2.** Identified DEGs between diabetic glomeruli and normal bladder.

**Additional file 3: Table S3.** Identified DEGs between diabetic glomeruli and normal glomeruli.

**Additional file 4: Table S4.** Identified DEGs between diabetic glomeruli and UTI-derived inflammatory cells.

**Additional file 5: Table S5.** Identified DEGs between diabetic glomeruli and bladder cancer.

**Additional file 6: Table S6.** Identified DEGs between diabetic tubules and normal bladder.

**Additional file 7: Table S7.** Identified DEGs between diabetic tubules and normal tubules.

**Additional file 8: Table S8.** Identified DEGs between diabetic tubules and UTI-derived inflammatory cells.

**Additional file 9: Table S9.** Identified DEGs between diabetic tubules and bladder cancer.

## Abbreviations

DKD: diabetic kidney disease
ESKD: end-stage kidney disease
MA: microalbuminuria
DM: diabetes mellitus
CKD: chronic kidney disease
UTI: urinary tract infection
BC: bladder cancer
LUT: lower urinary tract
DEG: differentially expressed gene
FC: fold change
qPCR: quantitative PCR
PPC: positive PCR control
RTC: reverse transcription control
OA: overt albuminuria
ACR: albumin-creatine ratio
eGFR: estimated glomerular filtration rate
HC: healthy control
FISH: fluorescent in situ hybridization
DAPI: 4′,6-diamidino-2-phenylindole
ROC: receiver operating characteristic
AUC: area under the curve
uBBOX1: urinary *BBOX1*
NAG: *N*-acetyl-beta-d-glucosaminidase
